# Diamond Sensor Technologies: From Multi Stimulus to Quantum

**DOI:** 10.3390/mi17010118

**Published:** 2026-01-16

**Authors:** Pak San Yip, Tiqing Zhao, Kefan Guo, Wenjun Liang, Ruihan Xu, Yi Zhang, Yang Lu

**Affiliations:** 1 Department of Mechanical Engineering, The University of Hong Kong, Hong Kong 999077, China; 2 Materials Innovation Institute for Life Sciences and Energy (MILES), HKU-Shenzhen Institute of Research and Innovation (HKU-SIRI), Shenzhen 518000, China

**Keywords:** diamond sensors, diamond MEMS, extreme environment sensing

## Abstract

This review explores the variety of diamond-based sensing applications, emphasizing their material properties, such as high Young’s modulus, thermal conductivity, wide bandgap, chemical stability, and radiation hardness. These diamond properties give excellent performance in mechanical, pressure, thermal, magnetic, optoelectronic, radiation, biosensing, quantum, and other applications. In vibration sensing, nano/poly/single-crystal diamond resonators operate from MHz to GHz frequencies, with high quality factor via CVD growth, diamond-on-insulator techniques, and ICP etching. Pressure sensing uses boron-doped piezoresistive, as well as capacitive and Fabry–Pérot readouts. Thermal sensing merges NV nanothermometry, single-crystal resonant thermometers, and resistive/diode sensors. Magnetic detection offers FeGa/Ti/diamond heterostructures, complementing NV. Optoelectronic applications utilize DUV photodiodes and color centers. Radiation detectors benefit from diamond’s neutron conversion capability. Biosensing leverages boron-doped diamond and hydrogen-terminated SGFETs, as well as gas targets such as NO_2_/NH_3_/H_2_ via surface transfer doping and Pd Schottky/MIS. Imaging uses AFM/NV probes and boron-doped diamond tips. Persistent challenges, such as grain boundary losses in nanocrystalline diamond, limited diamond-on-insulator bonding yield, high temperature interface degradation, humidity-dependent gas transduction, stabilization of hydrogen termination, near-surface nitrogen-vacancy noise, and the cost of high-quality single-crystal diamond, are being addressed through interface and surface chemistry control, catalytic/dielectric stack engineering, photonic integration, and scalable chemical vapor deposition routes. These advances are enabling integrated, high-reliability diamond sensors for extreme and quantum-enhanced applications.

## 1. Introduction

Diamond’s exceptional combination of mechanical rigidity, thermal conductivity, chemical inertness, wide bandgap, radiation hardness, and engineerable electronic and quantum defect properties has positioned it as a multipurpose material platform for sensing in extreme and interdisciplinary environments [1,2,3,4]. This review explores the wide range of diamond-enabled transduction, from high-Q mechanical resonators for vibration metrology [5,6] and pressure sensors based on piezoresistive, interferometric, and field emission mechanisms [1]. In thermal sensing, nitrogen-vacancy (NV) center thermometry is integrated with resonance-frequency and bolometric readout in single-crystal microelectromechanical system (MEMS) resonators [3,7]. Diamond further facilitates MEMS-based magnetic sensing, where integrated magnetostrictive films, which are frequently combined with piezoelectric layers, modulate the effective elastic modulus and thereby shift the resonant frequency under an external magnetic field [7]. Furthermore, diamond is utilized in deep-ultraviolet (UV, <230 nm) solar-blind optoelectronics and in color-center-based quantum photonic probes [3,4,8]. Its radiation detectors have also been validated in accelerator and fusion environments [9]. Diamond-based biosensors exploit the low fouling electrochemistry of boron-doped diamond (BDD) and the charge sensitive two-dimensional hole gas (2DHG) formed at hydrogen-terminated (H-terminated) surfaces [10,11,12]. Diamond gas sensors leverage surface transfer doping on H-terminated diamond (H-diamond), where adsorbates modulate the surface 2D hole channel. They also include catalytic Schottky/metal–insulator–semiconductor (MIS) stacks [10,11,13] and diamond imaging sensors, ranging from hardened AFM probes to chip-scale NV quantum microscopy [14,15,16]. Through various fabrication routes [17,18], diamond delivers high sensitivity, stability, and reliable operation under thermal, radiative, and corrosive conditions, while supporting pathways to on-chip integration and multi practicability [1,4]. The review also presents major challenges in diamond sensing, such as grain boundary and surface losses, limited wafer bonding yield and nanofabrication complications in single-crystal platforms, humidity and high-temperature stability of transfer-doped channels, interfacial degradation in magnetoelectric stacks, and coherence limits and surface induced noise for shallow NV centers. Advancing materials and device strategies, including hybrid interfaces, transition layer engineering, surface passivation, and photonic/microfabrication advances, are driving diamond sensors toward scalable, robust, and quantum-ready measurement systems [11,19,20,21,22].

## 2. Multi Stimulus

### 2.1. Vibration

Vibration sensing and high-frequency resonant technology are important in fields such as communication, quantum science, and industrial inspection. Diamond, with its extremely high Young’s modulus and low mechanical loss, has become a core choice in this field. In below Table 1, we compare three diamond types (nanocrystalline, single-crystalline, polycrystalline) and their trade-offs in performance, cost, and fabrication with silicon [23] and silicon carbide [24].

#### 2.1.1. Nanocrystalline Diamond (NCD) Resonators

NCD resonators fabricated using plasma-enhanced CVD (PECVD) or microwave plasma CVD (MPCVD) [25,26] provide a good balance of high frequency and moderate cost. However, they experience grain boundary losses.

Baldwin et al. in 2006 used PECVD to prepare 50–150 nm thick NCD films and fabricated a 2D weakly coupled ring resonator array [27]. The single ring resonator had a Q factor of 5000 at 40.18 MHz, and the tuning fork structure had a Q factor of >8000 at 37 MHz in the symmetrical bending mode.

Gaidarzhy et al. in 2007 grew NCD films by microwave plasma chemical vapor deposition (MPCVD) [28], adopting a coupled beam architecture, with a central beam and 20 cantilever units, each 500 nm wide and 300 nm long. The highest resonant frequency was 1.44 GHz, with a Q factor of 8660. The Q factors at 430 MHz and 630 MHz reached 21,500–23,200, which could be used for high-frequency mechanical memory.

Grain boundaries raise high-frequency energy loss, constraining performance compared to single-crystal devices.

#### 2.1.2. Single-Crystalline Diamond (SCD) Resonators

**Figure 1:**
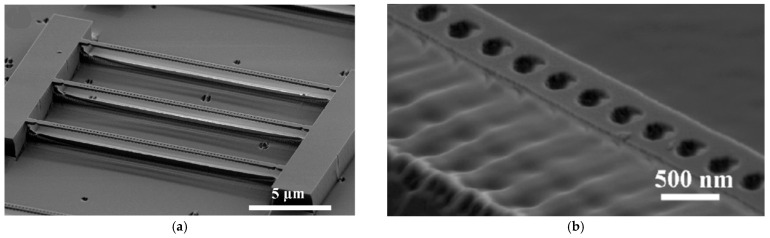

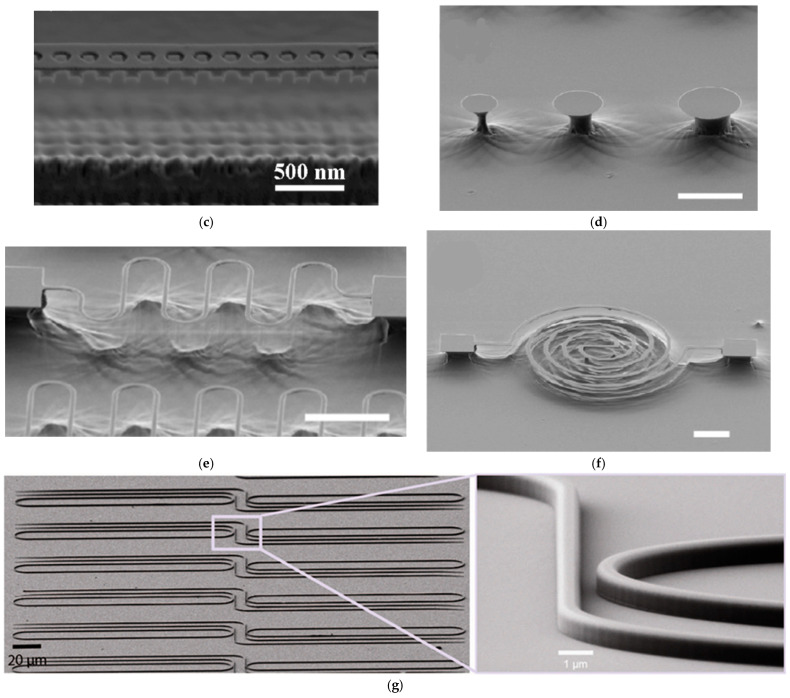
Representative structures and working mechanisms of diamond vibration sensor. (**a**–**c**) SEM images of (**a**) an array of fabricated diamond nanobeam cavity prototypes and (**b**,**c**) the cavity region of an individual nanobeam cavity [32]. (**d**–**f**) SEM images of (**d**) ~3–5 μm diameter undercut microdisks, (**e**) ~500 nm wide curved and (**f**) ~750 nm wide spiral nanobeams [32]. (**g**) SEM micrograph of an array of diamond racetrack resonators (330 μm length) coupled to diamond waveguides [34].

SCD resonators are normally fabricated using diamond-on-insulator (DOI) technology [29], Smart Cut [30,31], and anisotropic plasma etching [32], exhibiting ultra-high Q factors and quantum-level sensitivity, but with fabrication challenges [33]. Anisotropic plasma etching can be used to fabricate diamond microstructures, as shown in Figure 1a–f, but scaling up remains difficult. Ovartchaiyapong et al. in 2012 fabricated DOI membranes using the wafer bonding process [29], avoiding lattice damage caused by ion implantation and minimizing intrinsic SCD loss. By detecting the mode shape with a circular laser spot, the frequency stability of the asymmetric vibration mode was verified. At room temperature in vacuum, a cantilever with a length of 80 μm achieved a Q factor of 338,000, and it further increased at low temperature (10 K). Hausmann et al. achieved high-Q optical resonance in the near-infrared band based on a DOI platform, as shown in Figure 1g [34]. Furthermore, Tao et al. developed two preparation schemes, DOI and “Quartz Sandwich”, to fabricate cantilevers with quality factors exceeding one million [35]. The nitrogen doping concentration of electronic-grade SCD was less than 5 ppb, which can reduce defect-related loss. Through oxygen-termination or fluorine-termination surface treatment, surface damping was optimized, and the Q factor was highest for oxygen-terminated surfaces. As for application, Gu et al. used micromechanical resonators to detect the surface desorption properties of H-diamond [36]. They concluded that surface adsorbates reach saturation desorption at around 873 K, with a desorbed mass per unit area around 2.3 fg/μm^2^, corresponding to an equivalent thickness of approximately 1 nm. Nevertheless, it still faces challenges such as low DOI wafer bonding yield and complex nanofabrication processes.

#### 2.1.3. Polycrystalline Diamond (PCD) Resonators

PCD resonators are prepared using hot filament chemical vapor deposition (HFCVD) or PECVD processes. They have lower cost compared with SCD and are suitable for applications such as industrial-grade sensors and micro resonators focused on medium- and high-frequency vibration detection.

In 2005, Sepulveda et al. controlled the PECVD deposition parameters to achieve a Young’s modulus of 883–907 GPa for PCD [37], approaching but below the level of SCD. Under piezoelectric drive, the Q factor of 409 kHz reached 15,263. Bernstein et al. in 2015 used HFCVD to fabricate PCD hemispheres [38]. Hemispherical resonators with a diameter of 1.4 mm achieve a Q factor of up to 143,000 in the fundamental wineglass mode, with a ringdown time of 2.4 s in vacuum.

Furthermore, Zhou et al. designed a surface acoustic wave (SAW) resonator in 2013, with a structure of (002) AlN/Pt/PCD on silicon, enabling integration with other materials [39]. They used diamond’s large sound velocity to improve the velocity of a SAW and improved the interfacial boundary conditions with a Pt layer. Ultimately, they achieved a maximum SAW velocity of 13,656 m/s and an electromechanical coupling coefficient of 0.37%.

Diamond-based vibration sensors offer advantages in high-frequency, high-Q-factor, and harsh environments. The resonant noise is dominated by thermomechanical fluctuations, which are proportional to temperature and inversely related to the quality of the resonator and its mass. In practical applications, gas damping reduces the Q factor and increases noise, whereas dynamic desorption and adsorption of surface adsorbates introduce low-frequency noise, limiting the frequency stability. The nanocrystalline type focuses on high-frequency signal processing; the single-crystalline type tends to achieve quantum-level sensitivity detection, while the polycrystalline type pays more attention to cost and practicality. However, there are still some shortcomings. NCD grain boundaries increase high-frequency loss. The fabrication process of SCD is challenging, and the yield of wafer bonding in DOI still needs improvement. Although diamond’s intrinsic thermal stability is high under external constraints, the actual operating temperature is mainly limited by non-diamond components such as metal electrodes (e.g., Au, Pt), DOI bonding layer stability, and grain boundary oxidation. Consequently, the operational temperature is typically below diamond’s intrinsic thermal limit [40]. In the future, the material needs to be further optimized, and a “nanocrystal–single-crystal transition layer” diamond should be developed to balance cost and performance, achieving structural innovation and functional integration.

### 2.2. Pressure

Diamond, with its extremely high elastic modulus and tunable electrical/optical performance via doping and defects, has become a key material in the field of pressure sensing under extreme conditions. Diamond-based pressure sensors mainly include two technical approaches, piezoresistive and optical interferometric. The core difference lies in the signal transduction mechanism.

Nowadays, several related studies focus on piezoresistive pressure sensors. These sensors utilize the piezoresistive effect of diamond materials to transform signals by inducing a change in the resistance of the sensitive layer under pressure. This represents the mainstream technical approach for early diamond pressure sensors. In 1996, Chalker et al. designed piezoresistive pressure sensors [41], placing pressure-sensitive resistors at the edge (under tensile stress) and the center (under compressive stress of the diaphragm), using the longitudinal piezoresistive effect to improve response accuracy, and verified the high elasticity and chemical inertness of the diamond diaphragm, laying the foundation for the subsequent standardization of piezoresistive sensors. In 1998, Werner et al. systematically studied the piezoresistive effect of boron-doped PCD [42], reporting that the longitudinal piezoresistive effect is greater than the transverse piezoresistive effect, and proposed to preferentially adopt the longitudinal layout of pressure-sensitive resistors. In 2004, Yamamoto et al. used HFCVD to deposit undoped and boron-doped PCD on a silicon substrate [43], achieving a 30 μm linewidth pressure-sensitive resistor pattern through a metal mask and improving sensitivity by optimizing the membrane thickness and boron doping depth, which is suitable for medium–low-pressure scenarios from room temperature to 250 °C. In addition, Zaitsev et al. in 2001 used type IIa natural diamond anvils as the base [44]. Through high-temperature high-energy boron ion implantation and carbon ion irradiation, they constructed a p-i-p unipolar diode structure with the sensor having both pressure and temperature detection functions, which is suitable for high-pressure and high-temperature experimental devices such as diamond anvil cells. The intrinsic noise limit is mainly restricted by carrier thermal noise and flicker noise. Interface defects and uneven doping will exacerbate flicker noise, and this effect is particularly pronounced in the low-frequency range. In addition, under high-temperature conditions, the thermal fluctuations of the metal contact resistance will superimpose noise, resulting in drift in the pressure sensitivity.

In addition, there are two other types of sensors that do not rely on conductivity changes but achieve pressure detection through optical interference or field emission current changes, which are suitable for scenarios with requirements for anti-electromagnetic interference and high stability. Zhang et al. in 2014 designed and simulated a nano-diamond film pressure sensor based on the Fowler–Nordheim field emission equation [45], wherein pressure affects the electric field intensity (E = U/S) by changing the distance between the anode and cathode, thereby changing the emission current density. Salvatori in 2020 proposed an optical sensor based on Fabry–Pérot (FP) interference [46], used a 6 μm thick PCD membrane grown by MPCVD as the sensitive membrane, and formed a low-precision FP interference cavity with the end face of a single-mode optical fiber; by detecting the cavity length change caused by the micro deformation of the membrane induced by pressure, pressure measurement is achieved, with a range of 0–16.5 MPa, a sensitivity of 1 nm/kPa, and natural anti-electromagnetic interference, which is suitable for harsh environments with strong electromagnetism and high temperatures. Interferometric sensors rely on optical phase noise, which primarily stems from the linewidth of the light source and the shot noise of the detector. The actual limiting factors are as follows: environmental vibrations can undermine the stability of the interference cavity, and temperature fluctuations can cause thermal expansion of the cavity length, introducing additional phase noise; the non-uniformity of micro deformation in diamond films can exacerbate signal jitter.

### 2.3. Thermal Applications

Diamond-based temperature sensors leverage diamond’s physical, chemical, and quantum properties to demonstrate advantages over wide temperature ranges with high sensitivity and provide nanoscale spatial resolution in the case of NV center-based techniques.

**Figure 2:**
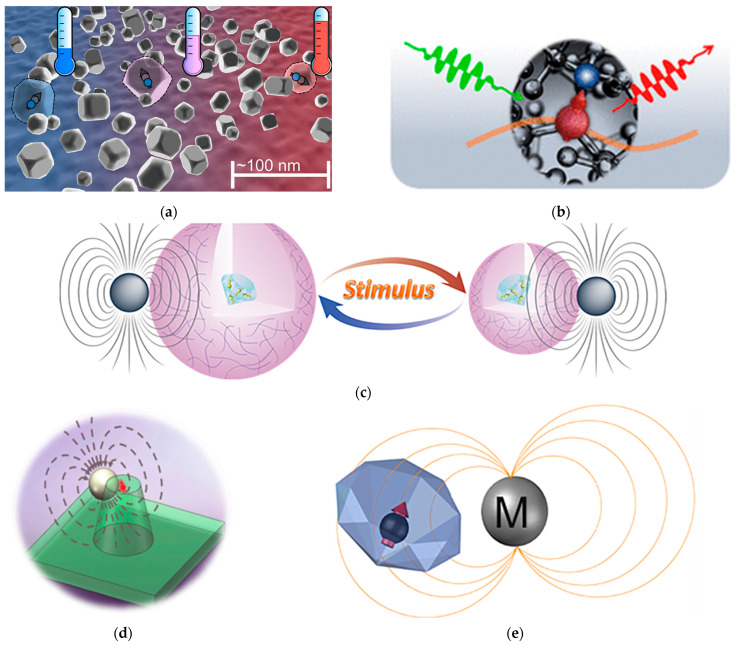
Mechanism of diamond thermal sensor based on NV centers. (**a**) Nanodiamonds containing single NV centers can serve as distributed probe temperature sensors [47]. (**b**) The NV center comprises a substitutional nitrogen atom and a nearby vacancy [48]. (**c**) Transducing mechanism of the hydrogel-based hybrid sensor [48]. (**d**) The hybrid nanothermometer comprises a single NV center in a diamond nanopillar and a nearby MNP [48]. (**e**) The hybrid nanothermometer comprises a nanodiamond with NV ensembles and a nearby magnetic nanoparticle [48].

There are advantages for NV centers to measure temperature at nanoscale spatial resolution, as atomic-scale defects can be placed near the target. However, the achievable resolution is limited by thermal diffusion and the NV sample standoff distance. Diamond NV thermometry utilizes the temperature dependence of the spin zero-field splitting (D) measured with optically detected magnetic resonance (ODMR). In 2013, Neumann et al. utilized the temperature-induced shift of D via ODMR to measure local temperature [47], as shown in Figure 2a. In 2023, Liu et al. adopted the thermomagnetic transducer in combination with pulsed ODMR protocols to convert temperature changes into magnetic signals detected by the NV center [48], achieving an improvement in sensitivity and meeting the requirements of extreme environments, such as high-temperature equipment and deep space exploration, as shown in Figure 2b–e.

The thermal resonant structure of SCD MEMS utilizes the change in its resonance frequency based on temperature to achieve temperature measurement. It is suitable for applications which require wide temperature operation and high resolution when multimode or auxiliary transduction is available. Zhao et al. in 2025 used the Smart Cut technology to fabricate a cantilever structure through high-quality SCD epitaxial layers and proposed a multimode resonant strategy [49], which broke through the response limit caused by the low-temperature coefficient of diamond, with a high sensitivity of about 22 nK/√Hz, a high-temperature resolution of 100 μK, and a wide temperature range from 6.5 to 380 K. Combined with high-frequency Q factors, it achieves a balance between high resolution and wide operational temperature range.

Device self-heating can cause failure, requiring real-time temperature monitoring. In the past, on-chip sensors and power diodes experienced electrical and thermal crosstalk. Lobaev et al. in 2025 achieved on-chip integration of power devices and diamond temperature sensors by using the temperature dependence of the forward voltage of the diamond sensor diode under fixed current to monitor the self-heating process of the power diode in real time [50], and with small electrical and thermal cross-talk between the diodes and low reverse leakage current, solving the response lag problem in traditional external sensors. It is applicable to thermal management scenarios of high-power electronic devices. It is worth noting that the upper limit of the actual operating temperature of the device depends on many factors, such as the conductivity stability of the boron-doped layer and the packaging interface [51].

### 2.4. Magnetic Applications

While diamond magnetometers using NV centers and optically detected magnetic resonance are widely investigated [52,53], diamond MEMS magnetic sensors provide a different scheme based on mechanical effects. The principles of diamond MEMS magnetic sensors are based on operation via Lorentz force mechanisms, magnetic torque force on a magnetized structure, or an effective Young’s modulus change by magnetic field (magnetostrictive ΔE effect) of the diamond MEMS resonator [54]. The merits of MEMS-based magnetic field sensors are their small dimensions, batch fabrication, flexibility in device design, and integration with electronics. Due to diamond’s high thermal conductivity, low thermal expansion, and mechanical robustness, diamond MEMS magnetic sensors integrated with magnetostrictive materials, which have high Curie temperatures, can overcome the weaknesses of Si-based MEMS with low reliability at elevated temperatures.

Zhang et al. in 2019 first used the Smart Cut method to fabricate SCD cantilever beams (60–160 μm length, 1.15 μm thickness) and deposited about 80 nm thick FeGa film by magnetron sputtering, forming a FeGa/SCD heterostructure [55]. The (110) preferred orientation of FeGa enabled a magnetoelectric ΔE response with a relative resonance frequency shift coefficient of about 75.5 ppm, a sensitivity of 4.83 Hz/mT at room temperature, a minimum detectable force of 2.14 × 10^−12^ N, and a theoretical minimum magnetic field resolution of about 142 pT. This work verified the core role of the ΔE effect in diamond MEMS magnetic field sensing.

The research group further verified the reliability of the FeGa/SCD structure under medium–high temperatures [56]. The sensitivity was 7.3 Hz/mT at 573 K, the magnetic noise level was about 4 nT/√Hz, and the minimum detectable magnetic field was about 159 pT under the specified averaging conditions. Through repeated heating–cooling cycle testing, the fractional resonance frequency fluctuation was <2.3 × 10^−6^, proving structural stability and meeting the requirements for industrial high-temperature magnetic field monitoring.

However, due to the large thermal expansion mismatch and interfacial adhesion mismatch between the polycrystalline sputtered FeGa and SCD, the FeGa/SCD MEMS composite resonator could only be operated up to around 573 K. By adding a buffered Ti layer between the FeGa thin film and diamond, enhancing interfacial adhesion and better managing thermal expansion mismatch, both the thermal stability and magnetic sensitivity of the diamond MEMS magnetic sensor were improved up to 773 K [57].

Furthermore, the magnetic sensitivity can be further improved by doping FeGa with boron, increasing magnetostriction and strengthening the ΔE effect, making the magnetic sensitivity as high as 152.1 Hz/mT [58].

Finally, they employed a Au/FeGa/Ti multifunctional electrode structure on SCD to achieve integration [59], which enabled the simultaneous realization of on-chip resonant excitation, magnetic field sensing, and electrical signal readout on the same FeGa film. It avoids signal interference from multiple-component integration. The diamond MEMS magnetic field sensors are based on the magnetostrictive ΔE effect. The sensitivity was improved by doping boron into the FeGa material; high temperature stability was enhanced by a Ti interface layer, and multichannel sensing through on-chip integration was realized through integrated electrode design. But it still faces problems such as interfacial degradation, metallurgical diffusion at extremely high temperatures, and the high cost of preparing thin SCD membranes [60]; the noise of the resonator is dominated by the resonator’s frequency and is related to the FeGa film. At high temperatures, the diffusion of the FeGa/diamond interface will reduce the magnetostrictive coefficient and increase noise; the release of film stress will exacerbate the resonator frequency drift and affect long-term stability.

### 2.5. Optoelectronics

The wide bandgap of approximately 5.47 eV, combined with high carrier mobilities, notable thermal conductivity and radiation durability, makes diamond an attractive material for optoelectronic sensing in difficult settings. These material performance indicators facilitate low dark currents, high breakdown fields, and reliable application over a large temperature range. Their band-to-band spectral response is confined to wavelengths shorter than approximately 225–230 nm, placing them in the deep UV region, underpinning solar-blind operation [61,62,63,64,65]. Advancements in high-purity single-crystal chemical vapor deposition (CVD) have resulted in transport performance that matches that of low-noise devices and deterministic control of color centers. This has broadened the scope of diamond-based sensors from traditional solar-blind photodiodes to nanoscale magnetometers and thermometers with optical readout [14,61,66].

**Figure 3:**
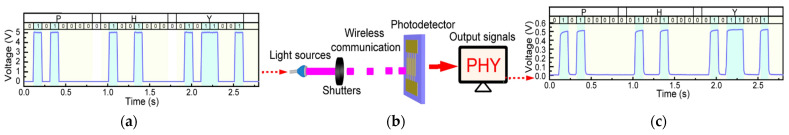
(**a**) Schematic illustration of the communication system. A broadband source emits solar-blind light (215–225 nm) filtered at 220 nm. A LabVIEW program generates TTL signals via an NI PCIe-6363 device to control a shutter, which transmits data encoded in ASCII. (**b**) TTL signal control of light modulation. The ASCII message is transferred into TTL signals where 5 V transfers to ‘1’ and opening the shutter and 0 V transfers to ‘0’ and closing the shutter, thus modulating the light beam to encode information. (**c**) Detection and data retrieval. A photodetector in series with a resistor senses transmitted light and transfers it to a voltage signal. A high voltage caused by increasing photocurrent corresponds to an open shutter (binary ‘1’) while a low voltage due to low photocurrent corresponds to a closed shutter (binary ‘0’). Monitoring these signals reconstructs the transmitted ASCII message, as shown by testing the transfer of ‘PHY’ with separators [62].

As shown in Figure 3, for classical diamond-based optoelectronic sensors, diamond’s large bandgap intrinsically suppresses visible response, yielding solar-blind deep UV photodetectors with excellent spectral selectivity without external filters [62]. Schottky and metal–semiconductor–metal (MSM) devices on CVD SCD exhibit high responsivity, low leakage, and fast response at elevated temperatures and under ionizing radiation, features that have been validated in space-borne instruments and accelerator environments [61,67,68]. Space missions have employed diamond UV detectors for stable, solar-blind radiometry with long-term radiation tolerance [67], while the CERN RD42 collaboration has demonstrated the radiation hardness and long-lasting functional properties of CVD diamond sensors under extreme conditions, establishing relevance for beam monitoring and dosimetry [68]. These demonstrations arise from the combination of wide bandgap, high intrinsic mobility, and strong lattice bonding, consistent with transport and photonic studies of high-purity diamond [2,61].

Beyond band-to-band photodetection, diamond hosts optically addressable point defects that function as optoelectronic sensors via spin-dependent photophysics [20,69,70]. The NV center enables ODMR for room-temperature sensing of magnetic fields, temperature, and electric fields, with well-established theoretical and experimental foundations supporting the defect’s spin dynamics and coupling pathways [3,12,71]. Practical modalities include scanning probe NV magnetometry for nanoscale magnetic imaging [70], nanoscale thermometry in living cells using single NV centers [7], and electric field sensing with single spins [72]. High sensitivity demands careful engineering of near-surface NV centers and reduction of surface-induced noise and decoherence. Complete explanation of these topics directs sensor design, implantation, and annealing protocols [71,73].

Continuing progress in diamond nanophotonics and materials engineering is advancing optoelectronic sensor performance by improving photon collection, spin coherence, and device integration. Photonic structures like solid immersion lenses, waveguides, and microcavities boost optical interface efficiency and emission collection for color center-based sensors, directly improving the signal to noise ratio in NV-based measurements [21,61,74]. In contrast, deep UV photodiodes benefit mainly from interface and device engineering. This includes anti-reflection coatings, transparent contacts, and optimized electrode geometries. Continuous advancements in CVD growth, surface termination, and depth-controlled implantation have enabled the reproducible creation of shallow low-noise color centers. These are suitable for chip-scale devices and can be heterogeneously integrated with microwave control, microfluidics, and CMOS platforms [21,71,73]. In addition, deep strained diamond may act as a highly efficient UV sensor [75]. Collectively, these developments advance compact, robust, and field-deployable diamond optoelectronic sensors for aerospace, industrial monitoring, biophotonics, and quantum-enabled metrology [61,67,70,71].

### 2.6. Radiation

Diamond’s high radiation toughness and low leakage current at room temperature make it a valuable material for radiation detection.

The RD42 collaboration directs research and progress into diamond radiation detectors, with a focus on their operation in high-energy physics experiments. In 1994, the RD42 collaboration group initiated the research and development of diamond detectors [76], aiming to provide radiation hard tracking and beam condition monitoring detectors for Large Hadron Collider (LHC) experiments, addressing issues such as short charge collection distance and immature preparation processes of early CVD diamond for both PCD and SCD by extending the charge collection distance, maturing growth and processing methods, and establishing a characterization and testing system for diamond detectors. In 2016, in response to the requirements of next-generation experiments such as the High-Luminosity LHC (HL-LHC) [77], the material supply chain was improved, the problem of signal attenuation under high particle flux was solved, and 3D diamond sensors were developed using laser-induced graphitic electrodes fabricated with ultrafast lasers.

In 2004, Edwards et al. proposed using PCD or SCD as the core to replace traditional silicon p-i-n diodes used for radiation dose monitoring in particle collision experiments such as BABAR [78], solving the problem of increased leakage current caused by radiation damage in silicon detectors and reducing signal distortion from temperature fluctuations in silicon detectors. By filling the trap energy level through the “radiation pumping” process, the charge collection efficiency can be raised to the saturation state. In the same year, Schmid et al. used fusion neutron spectroscopy for diamond measurement of 14 MeV fusion neutrons from D-T fusion in International Thermonuclear Experimental Reactor (ITER) diagnostics, with neutrons undergoing elastic scattering, inelastic scattering, and reactions to produce secondary particles [79]. By collecting the induced charge and performing pulse height spectroscopy, the neutron energy is resolved.

## 3. Biosensors

Research on biosensors has increasingly focused on stabilizing the electrode–electrolyte interface, as this contact often limits the long-term stability and accuracy of the readout. In practice, the durability of a signal depends as much on interfacial control as on the sophistication of the transduction step [80]. Various common electrode materials, such as noble metals and glassy carbon, can become fouled in biological fluids and exhibit potential drift. In contrast, BDD does not react adversely. Its predominant sp^3^-bonded network is robust enough to withstand aggressive media and provides a chemically robust, low-defect surface. This allows reliable redox reactions and direct electronic coupling in physiological solutions. In an aqueous environment, BDD offers a broad potential window for carbon electrodes, commonly ranging from 3.0 to 3.5 V depending on the electrolyte, termination, and microstructure. It also suppresses the formation of persistent polymeric films. These attributes shift the design challenge from generating signals to maintaining them in reactive biofluids.

The interfacial selectivity of diamond electrodes originates from controllable surface termination, which governs local charge and adsorption at the solid–liquid boundary. Hydrogen termination (H-termination) offers a hydrophobic surface with negative electron affinity. Because of surface conductivity and double-layer effects, it generally has higher interfacial capacitance and larger background currents, and it often enables faster outer sphere electron transfer kinetics. Oxygen termination introduces C=O and C-OH groups, providing a more hydrophilic, frequently negatively charged surface. At physiological pH, this can repel anions such as ascorbate while electrostatically favoring cationic analytes like protonated dopamine, enabling improved discrimination even when both are present. Yu et al. [81] have confirmed that termination tunes both adsorption energetics and electron transfer, producing distinct peak separations and potential shifts that have been used to detect dopamine at nanomolar levels in the presence of millimolar ascorbate. Beyond molecular selectivity, BDD exhibits notable resistance to surface fouling. Nonetheless, operation at very high anodic potentials or in the presence of strongly polymerizing analytes can still induce surface modification (including partial sp^2^ formation), so potential limits and surface pretreatment remain important.

**Figure 4:**
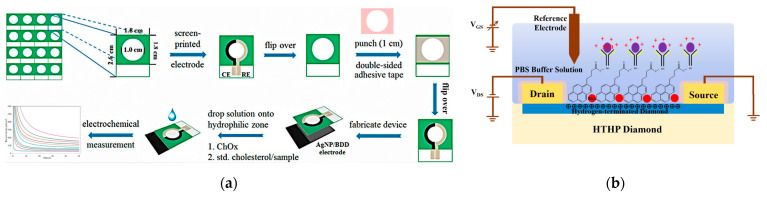
Representative architectures and working mechanisms of diamond-based biosensors. (**a**) Fabrication and operation process of a screen-printed Ag nanoparticles/BDD electrode for electrochemical cholesterol detection [83]. (**b**) Schematic of an H-diamond solution-gated field effect transistors (FET) functionalized with Pyr-NHS linkers for antibody-based toxin sensing [85].

These characteristics enable BDD to provide steady electrochemical feedback in undiluted serum and tissue fluids. Nanostructuring also improves performance by expanding the area and providing sites for bioreceptor immobilization. Enhanced charge transfer and robust chemistries for probe attachment are achieved by composites incorporating noble metal nanoparticles, such as gold, while largely maintaining diamond’s fouling resistance. However, integrating sp^2^-rich components, such as graphene [82] or carbon nanotubes, presents a drawback in that they diminish anti-fouling performance, and a narrower potential window may result with higher kinetics and increased surface area. Accordingly, as highlighted in Figure 4a, Gupta et al. [83] emphasized that morphology and composition should be matched to the dominant interfacial failure mode. For example, gold-decorated BDD can support aptamer- or antibody-based protein detection, whereas carefully engineered porous BDD structures can minimize interference during neurotransmitter sensing. Ficek et al. [84] reported that screen-printed diamond-based architectures extend these advantages to disposable formats, provided the inks and binders do not compromise the wide window and low fouling that make diamond attractive.

H-diamond extends the same material principles from electrochemical stability to charge-sensitive field effect operation. Beneath the hydrogenated surface of undoped or lightly doped diamond, a 2DHG forms via surface transfer doping, serving as a conductive channel located within nanometers of the solid–electrolyte interface. Variations in surface charge arising from ion adsorption or biomolecular binding modulate the local potential and hence the channel current. This direct solid–liquid coupling removes the need for a gate oxide at the sensing window, although device peripheries and metal contacts still require passivation to suppress leakage and parasitic faradaic currents.

Du et al. [85] detected staphylococcal enterotoxin B (SEB) with a linear range of 10^−15^–10^−7^ g/mL and a limit of detection of 1 fg/mL, achieving a sensitivity of −49.37 mV/lg of SEB concentration by using N-hydroxysuccinimide (NHS)-activated linkers to anchor antibodies in an oriented configuration shown in Figure 4b. The binding of positively charged toxins perturbs the electrical double layer, directly modulating the 2DHG carrier density and resulting in a detectable current variation. Zhang et al. [86] modified the H-terminated surface with gold nanoparticles. This enlarged the active sensing area and provided anchoring for thiolated probes, often enhancing sensitivity. Zou et al. [87] incorporated dielectric encapsulation and packaging to achieve further increased device-level reliability. This diminishes leakage and mechanical drift, demonstrating the importance of a balance between surface chemistry and electronic architecture for reliable sensing.

H-termination thus changes how the diamond surface behaves during sensing. A surface that functions as a chemically stable electrode in BDD-based electrochemistry becomes, in an H-diamond solution-gated FET, a charge-sensitive interface where small variations in interfacial potential immediately influence the channel current. Moving from BDD to H-diamond is therefore not a change in lattice structure but in doping level, surface chemistry, and conduction mechanism. BDD relies on bulk boron doping for redox-based measurements that benefit from a wide potential window and low fouling. H-terminated, undoped or lightly doped diamond relies on surface transfer doping to form a 2DHG that is exquisitely sensitive to interfacial charge. Diamond’s mechanical and chemical resilience support both modalities, enabling platforms that combine chemically selective electrochemistry with charge-sensitive field effect detection.

## 4. Quantum Sensors

Diamond’s NV center has become an emerging, versatile solid-state platform in quantum sensing applications. Due to diamond’s spin triplet ground state, its NV center can be optically initialized, optically read out via spin-dependent fluorescence, and coherently manipulated at or even above room temperature, allowing precise measurements of magnetic and electric fields, temperature, and pressure from the nanoscale to macroscopic areas via ODMR and related spin control protocols [53,71,88,89,90,91]. However, it is still affected by fluctuations in surface charges and background fluorescence noise. Different studies have investigated NV center-based magnetometry, which combines nanoscale spatial resolution with quantitative sensitivity. This technical capability fosters research applications across a wide range of condensed matter physics, materials science, imaging and biological systems [70,92,93,94,95,96,97,98,99,100,101,102]. Full-vector magnetometry typically requires measuring multiple NV orientations or rotating the sensor or sample to obtain projections along different crystallographic axes.

The well-characterized spin Hamiltonian shifts are fundamental to NV quantum sensing, including magnetic field Zeeman shifts for vector magnetometry and Stark effects for electric field detection. However, practical room-temperature sensitivity is limited and often affected by strain and local charge noise, especially near surfaces. Temperature- and pressure-dependent D is used for thermometry and high-pressure metrology. Decoupling temperature and stress or pressure contributions generally requires calibration or multiparameter protocols like double quantum sensing, which mitigate cross sensitivities. These effects have been demonstrated in single-defect and ensemble devices, including in diamond anvil cell environments [7,14,88,103].

Advancements in diamond growth techniques and coherent spin control have greatly improved sensitivity [104]. This is achieved by extending coherence times and boosting both spin control and photon collection efficiency. Bandwidth and dynamic range have been improved through protocol and readout engineering. This includes filter function design, lock-in and rapid scan techniques and widefield imaging [88,105,106]. Various technological platforms based on NV centers have been developed, including scanning probe tips, widefield quantum diamond microscope chips, and microfabricated or integrated photonic devices. These platforms enable a range of quantum-enhanced metrological techniques, such as double quantum protocols, ancilla-assisted repetitive readout (such as using 14N or 13C), and microwave-free detection modalities. These include relaxometry, operation near the ground-state level anticrossing (GSLAC) at approximately 102–103 mT (primarily sensitive to the axial field component with limited dynamic range), and all optical approaches.

NV centers have also been employed in nanoscale nuclear magnetic resonance (NMR) and spectroscopy applications, including the implementation of polarization transfer to external spin systems. Additional applications include imaging current distributions and spin textures in quantum materials and utilizing coupled electronic and nuclear spin dynamics within diamond to achieve gyroscopic sensing, typically leveraging the 14N or 13C nuclear spin as a long-lived ancilla to measure rotation-induced phase accumulation [107,108,109,110,111,112,113].

The NV center platform offers clear benefits and many applications. However, a number of practical challenges remain to be solved. These include shallow NV centers affected by induced noise from the diamond surface, such as fluctuating spins and charges, charge state instability, and limited photon collection efficiency. During recent years of technical advancement, better surface chemistry control and passivation strategies have been developed, and comprehensive engineering solutions in building diamond-based integrated photonic devices and nanophotonic structures have been shown to improve photon collection efficiency and scalability. These advancements collectively increase usable photon collection rates and make large-scale production more feasible [73,114,115,116]. Modern technological and methodological improvements in diamond NV centers have led to a stable and multifunctional quantum sensor platform operating at room temperature. In addition, classical microfabrication techniques (e.g., DOI, Smart Cut) support NV center array fabrication. The DOI process (detailed in Section 2.1.2) enables accurate thinning and bonding of SCD membranes, improving large-scale NV center implantation with uniform depth. Du et al. [101] developed a self-aligned patterning technique for fabricating high-performance diamond sensor arrays with nanoscale features, which facilitates scalable fabrication of diamond quantum sensors. Moreover, strain engineering of diamond as an emerging field will drive quantum application [117,118]. Continuing developments are concentrated on improving performance metrics and broadening application areas in the long run, driving diamond deployment across various physical, chemical, and biological measurement modalities [70,71,92].

## 5. Other Sensing Applications

### 5.1. Gas Sensors

**Figure 5:**
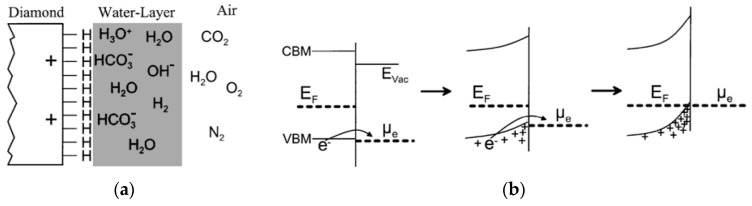
(**a**) A schematic diagram illustrates a H-diamond surface interacting with a water layer forming in air. This water layer establishes an electron system capable of functioning as a surface acceptor for the diamond. (**b**) An illustration shows how band bending changes during electron transfer at the diamond–water interface. The electron exchange between the diamond and water layer is controlled by a redox reaction [10].

Diamond is an attractive platform for gas sensing because it possesses excellent chemical inertness and thermal stability, and its surface electronic properties can be modulated by adsorbates. The common design of diamond-based gas sensors utilizes p-type surface conductivity that arises on H-diamond via surface transfer doping. In this process, electrons from the near-surface valence band are transferred to redox active species in an adsorbed aqueous layer under ambient humidity. This transfer is driven by Fermi level alignment with acceptor states in the layer and leaving holes in the diamond surface region. H-termination yields negative electron affinity. Under transfer doping conditions, the resulting surface potential causes upward band bending that facilitates near-surface hole accumulation. In dry air or vacuum, this surface hole channel can weaken substantially or collapse, though partial or transient conductivity can persist due to residual adventitious layers or other weak acceptors. As shown in Figure 5, changes in the composition and charge transfer characteristics of this interfacial aqueous/redox layer upon exposure to oxidizing or reducing gases can gate the near-surface hole channel, thereby modulating the device’s conductance [10,119,120,121]. Recent publications have comprehensively analyzed the physics and material aspects of this transfer doping mechanism, its sensitivity to adsorbates and its implications for sensor design. This research provides a fundamental understanding of the gas-induced conductivity changes on H-diamond surfaces [10,119,120].

Based on surface physics, hydrogenated diamond FETs and chemiresistors have been engineered to transduce gas adsorption into electrical signals for sensing and detection, with target chemical analytes including NO_2_ and NH_3_, as well as humidity effects. In these devices, the adsorbate-induced modification of the surface aqueous/redox layer acts as an effective chemical gate, producing measurable threshold voltage shifts or conductance changes at room temperature and above. Responses to NO_2_ (typically increasing conductance) and NH_3_ (typically decreasing conductance) depend strongly on ambient humidity because interactions often proceed via changes in the surface water film. Reversibility and kinetics are likewise humidity- and temperature-dependent and can be tuned by surface functionalization. Recovery from NO_2_ exposure can be slow at low humidity and may require thermal or UV assistance. Cross sensitivity to other oxidizers (such as O_3_) and the influence of mixed NO_x_ environments on response and recovery are relevant considerations for selectivity and calibration [122,123,124,125]. Broader reviews and topical overviews consolidate the operational regimes and transduction strategies across diamond-based device types and surface chemistries [124,126].

In addition to solely surface-conductive devices, metal/diamond Schottky and metal–insulator–diamond structures have also been investigated for hydrogen sensing by exploiting catalytic dissociation on metals such as Pd and subsequent modification of barrier properties, metal work function, interfacial dipoles, and charges/traps in the insulator at the diamond interface. The fast and reversible H_2_ response of Pd/diamond devices primarily stems from Pd’s catalytic dissociation/absorption of hydrogen and the associated formation of PdHx, which typically decreases the Pd work function and modulates barrier height. In MIS stacks, hydrogen can either introduce or passivate fixed charges and traps within the dielectric. This process shifts the thresholds and barriers independently of the diamond channel. Diamond’s wide bandgap and high thermal conductivity contribute to its stability, low leakage and ability to operate at high temperatures [127,128,129,130]. These complementary mechanisms facilitate the transfer-doped surface conduction found in H-terminated chemiresistors and FETs. This integration allows for sensing across a larger range of gases and operating circumstances, provided correctly chosen catalytic and oxide overlays are used to aim for exact reactions. However, cross sensitivities to factors like humidity and oxygen often remain [127,128,130]. Relevant studies of NCD platforms further opens sensing integration pathways and form factors. However, their conduction and surface chemistry, in addition to mixed terminations, sp^2^-rich grain boundaries and grain boundary adsorption, can differ from single-crystal H-diamond. This distinction may offer parallel conduction paths requiring specific adjustments and design strategies [131].

Diamond-based gas sensors provide obvious advantages and various uses due to intrinsic properties such as chemical robustness, radiation hardness, and compatibility with high-temperature application. For H-terminated surface channels, dehydration or oxidation can reduce the negative electron affinity and hinder the interfacial charge transfer needed for transfer doping. This diminishes stability at raised temperatures. High-temperature robustness is more frequent in Schottky/MIS structures and catalytically functionalized interfaces. Continued operation under high-temperature environments can also motivate Pd grain growth or oxidation and change dielectric properties, requiring device designs to reduce drift.

Challenges that remain include separating the roles of ambient water and specific adsorbates in transfer doping, improving the long-term stability of H-termination during cycling, achieving reproducible surface chemistries and passivation, and engineering selectivity without sacrificing sensitivity. Recent advances include the integration of stabilized ultrathin oxide gates engineered to preserve or controllably modulate the H-terminated channel, since some oxides can suppress transfer doping if they disrupt the interfacial chemistry; controlling the interfacial layer; application of catalytic overlayers to target specific gases; and the use of microelectromechanical heating to tune kinetics and aid recovery, with the caveat that localized heating can dehydrate or otherwise alter the surface chemistry [125,126,128,129,130]. The outstanding characteristics of diamond make it a promising candidate for gas sensors with high reliability and selectivity. Continued progress in surface science and device engineering is expected to help diamond sensors stand out in severe environments [66,126,130,131].

### 5.2. Imaging Sensors

Diamond, with its extremely high hardness, unique NV quantum properties, and controllable electrical properties, occupies an important position in the field of nanoscale detection and quantum sensing technologies.

Shibata et al. achieved the integration of diamond AFM probes with PZT films [132], solving the problems of insufficient hardness and wear of traditional silicon/silicon nitride probes, and endowing the probes with self-sensing and driving dual mode functions, it is suitable for large-scale integrated circuit (LSI) wafer detection and other requirements.

Maletinsky et al. in 2012 used low-energy nitrogen ion implantation to form near-surface NV centers at a depth of about 10 nm below the diamond surface [70], activating them at 800 °C for 2 h, and they fabricated 200 nm diameter and 1 μm length nanocolumns through electron beam lithography and reactive ion etching, integrating them at the tip of the AFM probe, giving it nanoscale quantum sensing capabilities. Glenn et al. in 2015 fabricated diamond chips through CVD, ion implantation and annealing [133], using the magnetic sensing characteristics of diamond NV centers to achieve single-cell-level, widefield, and low background detection of immunomagnetically labeled cells, which verified the feasibility of the technology in quantitative biomarker quantification and complex sample analysis. As for practical application, Shaji et al. developed an integrated portable device using diamond quantum technology. This device allows on-site nanoscale magnetic imaging [134], overcoming the volume and environmental limitations of traditional equipment. It provides a practical method for field geological exploration and on-site industrial quality inspection. Potocký et al. reported a developed low-temperature CVD process in 2025 for preparing boron-doped nano-diamond (BNCD) coatings, which were used for self-sensing AFM probes [135], achieving conducting and self-sensing dual mode functions for the probes, with a spatial resolution of less than 50 nm.

Devices based on diamond have gradually evolved from laboratory technologies to practical application fields, such as large-scale integrated circuit testing, biomedical analysis, and geological exploration.

## 6. Summary and Future Roadmap

Diamond’s mechanical stiffness, low loss, wide bandgap, thermal conductivity, chemical inertness, and optically addressable defects enable integrated sensing across extremes of frequency, temperature, radiation, and scale. NCD/PCD/SCD resonators deliver high-frequency, high-Q mechanical sensors. SCD devices support quantum opto- and spin mechanics, while PCD films scale for harsh-environment MEMS. Pressure sensing employs boron-doped piezoresistive gauges/bridges in diamond diaphragms and optical interferometry, extending operation to high temperature and pressure. Thermal sensing spans nanoscale NV thermometry and resonant SCD MEMS with high Q and low loss, with noise floors set by transduction and readout. Diamond MEMS magnetic sensors integrating magnetostrictive films target high sensitivity and high-temperature reliability via interface engineering. In optoelectronics, solar-blind deep UV photodiodes show low leakage and radiation hardness. Radiation detectors for high-energy physics confirm radiation hardness and fast charge collection. Biosensing combines BDD’s antifouling electrochemistry with H-terminated FETs for label-free, real-time detection, and color center quantum optosensors are demonstrated. Gas sensors use H-terminated surface transfer doping for chemiresistors/FETs and Pd-based Schottky/MIS stacks for fast H_2_ response, with selectivity and drift set by humidity and surface stability. Diamond’s hardness and defects underpin imaging probes and widefield quantum microscopes. Shared advantages, such as high Q, low dark current, radiation/thermal robustness, and, for color centers, ODMR-enabled quantum readout, are moving diamond sensors toward field deployment.

There are still some system-level bottlenecks for sensors moving from laboratory prototypes to practical applications. These bottlenecks mainly lie in four key aspects: firstly, there are issues related to integration compatibility, such as signal loss at the interface when integrating diamond devices heterogeneously; secondly, there are limitations in environmental adaptability, with non-diamond components (such as metal leads, packaging materials, functional layers) becoming the bottleneck for the system’s operating temperature; thirdly, there are challenges in mass production consistency, with the volatility of diamond growth and micro–nano processing techniques making it difficult to control device batches; fourthly, there are problems in cost control, including high material costs and complex processing costs.

The future can break through these system-level bottlenecks through the following technical paths: for the demand for integration compatibility, impedance matching transition layers to reduce signal loss at the heterogeneous integration interface should be developed; for extreme environment adaptability, high-temperature-resistant and stable material systems such as platinum-based leads and ceramic packaging can be adopted to enhance the reliability of the entire device chain; a closed-loop control scheme for chemical vapor deposition growth and a collaborative optimization plan for high-precision lithography processes should be studied to ensure production consistency; for the cost control target, a low-cost technical route of replacing single-crystal diamond with polycrystalline diamond should be proposed. In addition, many alternative strategies can be adopted; a variety of emerging magnetostrictive materials have demonstrated superior performance in specific scenarios. For instance, shape memory alloys can be controlled by magnetic fields to restore their shapes, enabling the integrated function of “sensing-driving.” In addition to the traditional NV centers, the spectra of SiV^−^ centers are located in the near-infrared band (~738 nm), with a deeper penetration depth, making them suitable for deep magnetic imaging of biological tissues. The neutral nitrogen vacancy (NV^0^) centers are less affected by surface charge noise and are suitable for near-surface nanoscale temperature measurement.

## Figures and Tables

**Table 1:** Quantitative comparison of key sensor technologies with different materials.

Material	Q Factor	Sensitivity	Cost (Relative)	Key Limitations
NCD	23200	Low-Moderate	Low to Medium	Grain boundary losses
SCD	10^7^	High	High	Hard to fabricate
PCD	143,000	Moderate	Low to Medium	Grain boundary losses
Silicon	140,000	Moderate	Low	Low thermal stability
SiC	10^7^	High	Medium to High	High processing cost

## Data Availability

No new data were created or analyzed in this study.
